# The *Panax ginseng* Berry Extract and Soluble Whey Protein Hydrolysate Mixture Ameliorates Sarcopenia-Related Muscular Deterioration in Aged Mice

**DOI:** 10.3390/nu14040799

**Published:** 2022-02-14

**Authors:** Min-Ji Han, Seok-Jun Park, Sang-Jun Lee, Se-Young Choung

**Affiliations:** 1 Department of Life and Nanopharmaceutical Sciences, Graduate School, Kyung Hee University, 26, Kyungheedae-ro, Dongdaemun-gu, Seoul 02447, Korea; hanmj_p3@khu.ac.kr; 2 Health & Nutrition R&D Group, Maeil Dairies Co., Ltd., Pyeongtaek 17714, Korea; sj.park@maeil.com; 3 Holistic Bio Co., Seongnam 13494, Korea; leesjun@holistic-bio.com; 4 Department of Preventive Pharmacy and Toxicology, College of Pharmacy, Kyung Hee University, 26, Kyungheedae-ro, Dongdaemun-gu, Seoul 02447, Korea

**Keywords:** sarcopenia, *Panax ginseng* berry extract, soluble whey protein hydrolysate, muscular deterioration, aging, skeletal muscle, protein turnover

## Abstract

Sarcopenia is prevalent as the aging population grows. Therefore, the need for supplements for the elderly is increasing. This study aimed to investigate the efficacy and mechanism of a *Panax ginseng* berry extract (GBE) and soluble whey protein hydrolysate (WPH) mixture on a sarcopenia-related muscular deterioration in aged mice. Ten-month-old male C57BL/6J mice were administered three different doses of the GBE + WPH mixture for 8 weeks; 700 mg/kg, 900 mg/kg, and 1100 mg/kg. Grip strength, serum inflammatory cytokines level, and mass of muscle tissues were estimated. The deteriorating function of aging muscle was investigated via protein or gene expression. Grip strength and mass of three muscle tissues were increased significantly in a dose-dependent manner, and increased anti-inflammatory cytokine alleviated systemic inflammatory state. The mixture resolved the imbalance of muscle protein turnover through activation of the PI3K/Akt pathway and increased gene expression of the muscle regeneration-related factors, while decreasing myostatin, which interferes with muscle protein synthesis and regeneration. Furthermore, we confirmed that increased mitochondria number in muscle with the improvement of mitochondrial biogenesis. These physiological changes were similar to the effects of exercise.

## 1. Introduction

Sarcopenia, described as a progressive decline in skeletal muscle mass, strength, and function with advanced age, is prevalent nowadays, as the aging population increases [1]. Physical inactivity, declined hormone secretion, and a decrease in protein intake exacerbate sarcopenia in the elderly [2]. In addition, diseases such as cardiovascular disease, dementia, and diabetes mellitus increase the incidence of sarcopenia [3].

The chronic low-grade inflammation, one of the age-related systemic changes, is responsible for loss of muscle mass [4]. Elevated pro-inflammatory cytokines with aging, such as interleukin-6 (IL-6) and tumor necrosis factor-alpha (TNF-α), degrade skeletal muscle protein, resulting in decreased muscle mass and strength [5]. Meanwhile, the anti-inflammatory cytokine interleukin-10 (IL-10) suppresses the pro-inflammatory response and improves insulin sensitivity and mitochondrial biogenesis in aged skeletal muscle [4,6]. Serum IL-10 level increases slightly in elderly sarcopenia, but it is considered to be a compensation for the chronic low-level inflammatory conditions [7]. Therefore, the IL-6/IL-10 ratio is being used as a reliable marker to judge the inflammatory status, and it practically tends to increase in elderly sarcopenia patients [7,8,9].

Aging-related metabolic changes in skeletal muscle, such as impaired muscle protein turnover, mitochondrial capacity, and muscle regenerative capacity, provokes sarcopenia [10]. Phosphatidylinositol-3-kinase (PI3K)/Akt (protein kinase B) pathway is an important signaling pathway for maintaining the balance between muscle protein synthesis and degradation [11]. When this pathway is activated by insulin-like growth factor-1 (IGF-1) or insulin, muscle protein synthesis increases via up-regulating ribosomal protein S6 kinase 1 (S6K1) and eukaryotic translation initiation factor 4E-binding protein 1 (4E-BP1), and muscle protein degradation decreases via down-regulating muscle atrophy F-box protein 32 (Atrogin-1) and muscle ring finger-1 (MuRF1), which are E3 ubiquitin ligases. With aging, the PI3K/Akt pathway activity is diminished as the secretion of growth hormones such as IGF-1 gradually decreases [10]. Pro-inflammatory cytokines, IL-6 and TNF-α, also disrupt this signaling pathway directly or indirectly through the c-Jun N-terminal kinase (JUN) or Nuclear factor-κB (NF-κB) pathway, promoting the breakdown of muscle protein [12].

Mitochondria are the prominent energy-producing organelles in skeletal muscle, so that they are an important factor that determines muscle function and strength [13]. However, they decline in number and function with aging, resulting in increased reactive oxygen species (ROS) production and oxidative stress, which activates forkhead box O3 (FoxO3) in the PI3K/Akt pathway, leading to increased transcription of E3 ubiquitin ligases as well as an inflammatory response [10,14]. In terms of muscle fiber regeneration, muscle satellite cells regenerate skeletal muscles in the order of proliferation, differentiation, and fusion. However, in aging muscles, the activity and function of the muscle satellite cells rapidly decreases, resulting in diminished muscular regenerative capacity [13,15,16].

Exercise is usually prescribed as a remedy for sarcopenia because aerobic exercise alleviates mitochondria-derived problems with aging, while resistance exercise increases muscle mass and improves muscle function [17]. However, this is challenging for older people who have mobility difficulties or are unable to move. Thus, supplements preventing sarcopenia are needed for them. In a previous study, we confirmed the synergetic effect of a GBE and WPH mixture as a supplement for muscle atrophy in the immobilization (IM)-induced muscle atrophy mouse model. We also found the optimal mixing ratio comparing three different mixing ratios of GBE and WPH [18]. Therefore, in this study, we aimed to investigate whether the GBE and WPH mixture also effective in 10-months old mice and to examine aging-related mechanism changes in the skeletal muscle.

## 2. Materials and Methods

### 2.1. Preparation of GBE and WPH

The GBE and WPH mixture preparation were performed as previously described [18]. Briefly, whey protein concentrate was dissolved in distilled water to a 20% concentration and then pH was adjusted from 7 to 7.5. Next, three enzymes; Alcalase L FG (Novozyme, Denmark), Protamex (Novozyme, Denmark), and Flavourzyme 1000 L (Novozyme, Denmark) were added to the solution and incubated, followed by inactivation, filtration, and spray drying. GBE was extracted with water under reflux condition, then filtered, evaporated, and spray dried. Neocremar Co., Ltd. (Seoul, Korea) provided WPH and Holistic Bio Co. (Seongnam, Korea) provided GBE. Powdered WPH and GBE were mixed in an 8:1 ratio and dissolved in 0.5% carboxymethylcellulose according to the dose of each administration group.

### 2.2. Mice and Design of Experiment

Ten-month-old male C57BL/6J mice (Janvier labs, Saint-Berthevin, France) were housed in Kyung Hee University’s animal facility maintained under a 12 h:12 h light-dark cycle at constant temperature (25 ± 1 °C). Normal diet (rodent diet with 10% kcal% fat; D12450B) and water were available ad libitum. Mice were adapted to the environment for a week and divided into the control group and the GBE + WPH mixture administration groups (*n* = 10 per group); L group (700 mg/kg GBE + WPH mixture), M group (900 mg/kg GBE + WPH mixture), and H group (1100 mg/kg GBE + WPH mixture). The ratio of WPH to GBE in the mixture is 8:1, which was selected as the optimal ratio following a previous study [18]. Oral administration was conducted for 8 weeks, and we measured body weight and grip strength twice a week during the experiment. To measure the grip strength, the mouse was placed on the grid connected to the grip strength meter (Bioseb, Chaville, France) and their tail was pulled while the mouse grasped the grid. The average grip strength per week was normalized by body weight. After sacrifice, we collected blood samples and leg muscle tissues (quadriceps, gastrocnemius, and soleus). The Institutional Animal Care and Use Committee guidelines of Kyung Hee University approved the animal study, and the approval number is KHSASP-20-383.

### 2.3. Assessment of Serum Cytokine Levels

We used serum obtained from the inferior vena cava of the mice. Serum IL-6 and TNF-α concentrations were assessed using immunoassay kit (R&D system, Minneapolis, MN, USA) and anti-inflammatory cytokine IL-10 level was measured using EliKine™ mouse IL-10 ELISA kit (Abbkine, Wuhan, China). We conducted assessment according to the protocol provided by each supplier using an ELISA microplate reader (Bio-Tek Instruments Inc., Vermont, WI, USA).

### 2.4. Histological Analysis

Gastrocnemius was used for histological analysis. It was fixed in 4% paraformaldehyde, embedded in paraffin, and sliced into 5 µm sections. Hematoxylin and eosin (H&E) staining was conducted for 13 h, and cross-sectional area (CSA) images were taken using an optical microscope (Olympus, Tokyo, Japan) with 400× magnification. Image J software (National Institute of Health, Bethesda, MD, USA) was used for quantification of myofibers CSA.

### 2.5. Western Blotting

Frozen gastrocnemius muscle tissue was ground into small pieces in liquid nitrogen and lysed in a lysis buffer containing cOmplete™ Protease Inhibitor Cocktail and PhosSTOP™ (Roche Diagnostics, Indianapolis, IN, USA), and then centrifuged. A Pierce™ BCA Protein Assay Kit (Thermo Fisher Scientific, Rockford, IL, USA) was used to measure the protein concentration and same concentration of protein of each group was subjected to SDS-PAGE. After transferring, membranes were blocked by 5% skim milk and incubated with a primary antibody overnight. The primary antibodies used for Western blot were p-Akt, Akt, p-mTORc1, mTORc1, p-S6K1, S6K1, p-4E-BP1, 4E-BP1, p-FoxO3a, and FoxO3a (Cell signaling, MA, USA), MuRF1 and Atrogin-1 (Santa Cruz Biotechnology, Santa Cruz, CA, USA), and β-actin (GeneTex, Irvine, CA, USA). The next day, the membranes were incubated with the corresponding secondary antibodies for visualizing the protein bands using LAS3000 luminescent image analyzer (Fuji Film, Tokyo, Japan). β-actin was used as a loading control and Image J software (National Institute of Health, Bethesda, MD, USA) was used for quantification.

### 2.6. Quantitative Real Time-PCR (qRT-PCR)

We homogenized gastrocnemius tissues in liquid nitrogen and an easy-RED™ (iNtRON, Seongnam, Korea) was used for RNA extraction. cDNA synthesis was performed using a PrimeScript™ 1st strand cDNA Synthesis Kit (TaKaRa, Tokyo, Japan) according to the manufacturer’s protocol. qRT-PCR was conducted using TB Green™ Premix Ex Taq™ (TaKaRa, Tokyo, Japan) and a Step One Plus™ Real-Time PCR System (Applied Biosystems, Foster City, CA, USA). The primer sequences were presented in Table 1. The gene expression levels were normalized to β-actin, and the 2^−ΔΔCT^ method was used to calculate the relative expression level. For measurement of mitochondrial DNA (mtDNA) content, AccuPrep^®^ Genomic DNA Extraction Kit (Bioneer, Daejeon, Korea) was used for total DNA isolation according to the manufacturer’s instruction. 16S rRNA and Hexokinase 2 were used for quantification of mitochondrial and nuclear genomes, respectively, and qRT-PCR was performed identically to that described above.

### 2.7. Statistics

Statistical analysis was performed using a one-way ANOVA, followed by Tukey’s test and the data were presented as mean and standard deviation (SD). Statistical significance was calculated using SPSS version 25 statistical software (IBM, Chicago, IL, USA). The significance was represented as * *p* < 0.05, ** *p* < 0.01, and *** *p* < 0.001 compared to the control group.

## 3. Results

### 3.1. The GBE + WPH Mixture Alleviates the Chronic Low-Grade Inflammation in Aged Mice

**Figure 1:**
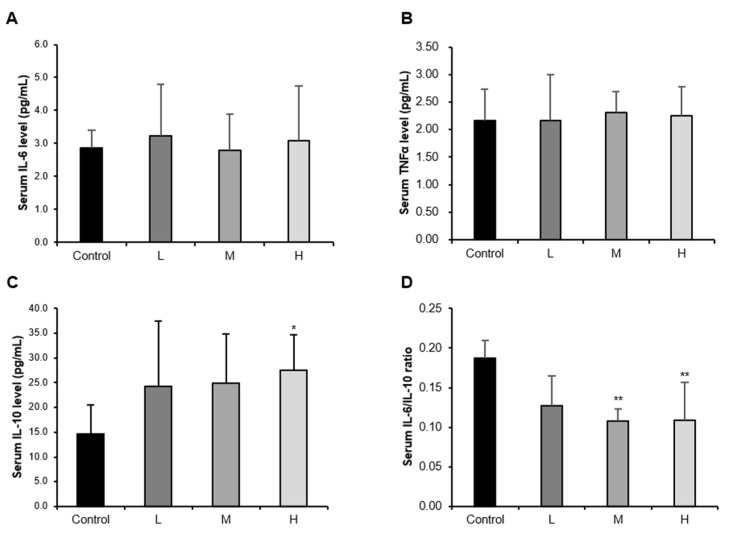
Effect of the GBE + WPH mixture on serum cytokine levels in aged mice. Ten-month-old male C57BL/6 mice were administered three different doses of the GBE + WPH mixture for 8 weeks; 700 mg/kg, 900 mg/kg, and 1100 mg/kg. (**A**) Serum IL-6 level measured by ELISA. (**B**) Serum TNF-α level measured by ELISA. (**C**) Serum IL-10 level measured by ELISA. (**D**) Serum IL-6/IL-10 ratio. The data are shown as mean ± SD. * *p* < 0.05, ** *p* < 0.01 versus control.

In the elderly, an increased ratio of pro-inflammatory to anti-inflammatory cytokines is characterized by a chronic low-grade inflammatory response [7]. Skeletal muscle has been shown to be a potent regulator of the immune system because it produces and releases cytokines, such as IL-6, IL-7, IL-15, etc., which are called myokines [19]. Therefore, a link between sarcopenia and immune senescence has recently been raised [20]. We measured serum pro- and anti-inflammatory cytokine levels and examined the change in the ratio. As a result, there was no significant difference in pro-inflammatory cytokines IL-6 and TNF-α in the GBE + WPH mixture administered groups compared to the control group (Figure 1A,B). In contrast, serum IL-10 levels were elevated in a GBE + WPH dose-dependent manner so that the IL-6/IL-10 ratio, a marker for judging the inflammatory status, was significantly reduced in the M and H groups (Figure 1C,D).

### 3.2. The GBE + WPH Mixture Suppresses Muscle Weakness and Increases Muscle Mass in Aged Mice

**Figure 2:**
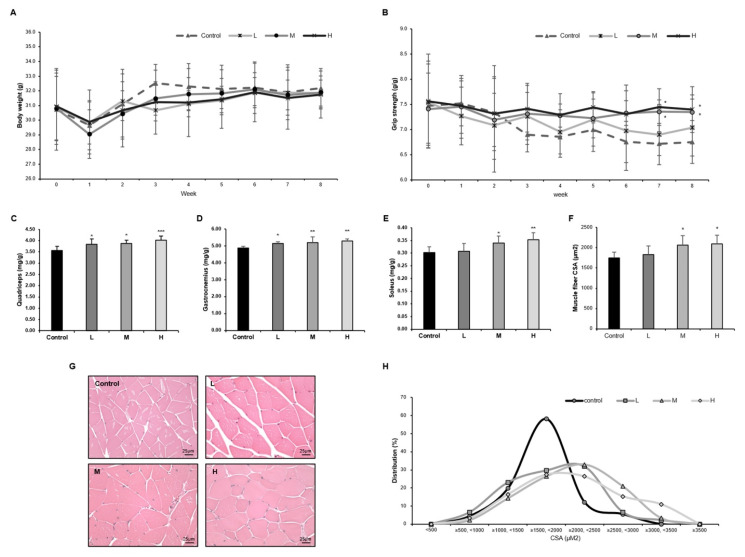
Effect of the GBE + WPH mixture on muscle atrophy in aged mice. Ten-month-old male C57BL/6 mice were administered three different doses of the GBE + WPH mixture for 8 weeks; 700 mg/kg, 900 mg/kg, and 1100 mg/kg. (**A**) Body weight curves. (**B**) Grip strength curves. (**C**) The weight of quadriceps muscle tissue. (**D**) The weight of gastrocnemius muscle tissue. (**E**) The weight of soleus muscle tissue. (**F**) The mean cross-sectional area (CSA) of each muscle fiber. (**G**) H&E staining of gastrocnemius. (**H**) The distribution graph of muscle fiber CSA. Scale bars 25 µm. The data are shown as mean ± SD. * *p* < 0.05, ** *p* < 0.01, *** *p* < 0.001 versus control.

We investigated how effective the GBE + WPH mixture was in muscular strength and muscle mass. We measured body weight and grip strength twice a week for 8 weeks. There were no significant differences in body weight between groups at the end of the experiment (Figure 2A). The grip strength of the control group continued to decrease for 8 weeks, whereas the groups M and H administered with GBE + WPH continued to maintain the grip strength, showing a significant difference compared to the control group (Figure 2B). Furthermore, the mass of fast-twitch fiber muscle (quadriceps and gastrocnemius) and slow-twitch fiber muscle (soleus) was dose-dependently increased (Figure 2C–E). The cross-sectional area (CSA) of the gastrocnemius muscle was also increased with the same tendency as muscle mass (Figure 2F). In the distribution graph of CSA, muscle fibers of the control group were concentrated between 1500 and 2000 μM^2^, but the GBE + WPH administered groups showed an increase of more than 2000 μM^2^ of the muscle fibers and CSA was widely distributed (Figure 2G,H).

### 3.3. The GBE + WPH Mixture Activates PI3K/Akt Pathway in Skeletal Muscle of Aged Mice

**Figure 3:**
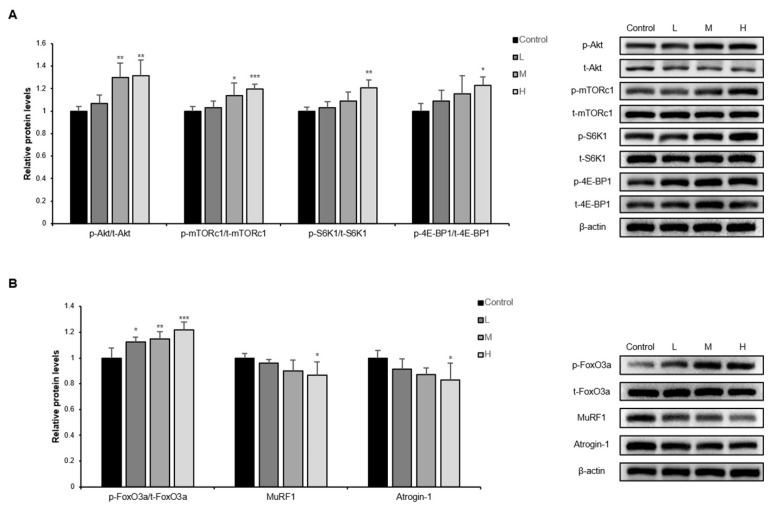
Effect of the GBE + WPH mixture on muscle protein synthesis and degradation mechanisms. Ten-month-old male C57BL/6 mice were administered three different doses of the GBE + WPH mixture for 8 weeks; 700 mg/kg, 900 mg/kg, and 1100 mg/kg. (**A**) Relative protein expression of factors related to muscle protein synthesis (p-Akt/t-Akt, p-mTORc1/t-mTORc1, p-S6K1/t-S6K1, and p-4E-BP1/t-4E-BP1) and Western blot images. (**B**) Relative protein expression of factors related to muscle protein degradation (p-FoxO3a/t-FoxO3a, MuRF1, and Atrogin-1) and Western blot images. The protein expression levels were normalized to β-actin level. The data are shown as mean ± SD. * *p* < 0.05, ** *p* < 0.01, *** *p* < 0.001 versus control.

Activation of the PI3K/Akt pathway induces muscle hypertrophy and inhibits muscle atrophy through regulating muscle protein turnover [11]. In the PI3K/Akt pathway, the muscle protein synthetic factors are phosphorylated in the order of PI3K, Akt, mammalian target of rapamycin complex 1 (mTORc1), S6K1, and 4E-BP1. The phosphorylation form of these factors is the active form. Thus, the phosphorylation ratio was measured by protein expression. The ratio increased in the GBE + WPH mixture administered groups dose-dependently, and the phosphorylation ratio of S6K1 and 4E-BP1, which are the lowest two factors on the synthesis side of the PI3K/Akt pathway, showed significant differences in the H group (Figure 3A). In terms of muscle protein degradation side, as the phosphorylation ratio of forkhead box O3a (FoxO3a) increased by the GBE + WPH mixture, the expression of FoxO3a’s sub-factors, Atrogin-1 and MuRF1, was significantly decreased in the H group due to phosphorylated FoxO3a localization from the nucleus to cytosol (Figure 3B).

### 3.4. The GBE + WPH Mixture Improves Muscle Regeneration and Mitochondrial Biogenesis in Skeletal Muscle of Aged Mice

**Figure 4:**
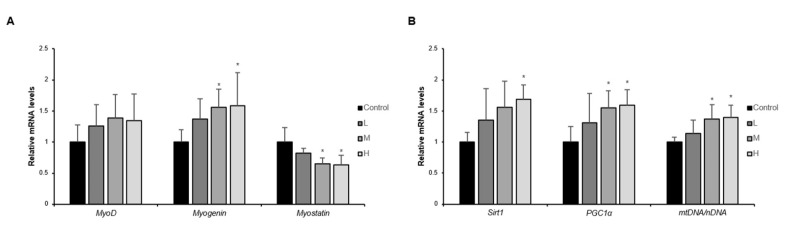
Effect of the GBE + WPH mixture on muscle regeneration and mitochondrial biogenesis. Ten-month-old male C57BL/6 mice were administered three different doses of the GBE + WPH mixture for 8 weeks; 700 mg/kg, 900 mg/kg, and 1100 mg/kg. (**A**) Relative mRNA expression of factors related to muscle regeneration (Myostatin, Myogenin, and MyoD). (**B**) Relative mRNA expression of factors related to mitochondrial biogenesis (Sirt1, PGC1a, and mtDNA/nDNA). The gene expression levels were normalized to β-actin level. The data are shown as mean ± SD. * *p* < 0.05 versus control.

The capacity of muscle regeneration reduces in the elderly due to the physiological activity of muscle stem cells declining with advancing age, leading to slow recovery after injury and development of sarcopenia [21]. MyoD and Myogenin are myogenic regulatory factors that increase, respectively in the early and late stages of differentiation. On the contrary, Myostatin, a myokine that suppresses muscle growth, restrains muscle differentiation via repressing MyoD and Myogenin expression. As a result of measuring gene expression of these factors, MyoD and Myogenin increased, and Myostatin decreased in the GBE + WPH mixture in a dose-dependent manner, showing similar efficacy in the M and H groups (Figure 4A). NAD-dependent deacetylase sirtuin-1 (Sirt1) is an NAD+-sensitive deacetylase with an essential role in metabolic regulation and is especially known to prolong life span and delay aging [22]. PGC1α is deacetylated by Sirt1, leading to its nuclear localization and increased mitochondrial biogenesis-related gene expression. As the gene expressions of Sirt1 and PGC1α improved by the GBE + WPH administration, the mtDNA/nDNA ratio also increased, confirming that the number of mitochondria was increased (Figure 4B).

## 4. Discussion

We previously conducted several studies using ginseng berry extract (GBE) and whey protein hydrolysate (WPH) to invent an effective supplement for sarcopenia. In the prior study, we selected the most effective WPH among four types of WPH [23], and in another study, we confirmed the synergetic effect of WPH and GBE mixture and established an appropriate dose using IM-induced muscle atrophy mouse model [18]. Therefore, this study aimed to investigate the effect of the GBE + WPH mixture on aging-related mechanisms in skeletal muscle and to confirm the dose-dependent effects using the aging mouse model that is clinically closest to sarcopenia. Furthermore, because we used the aging mouse model in this study, we measured not only muscular-related factors but factors that deteriorated by aging, such as chronic low-grade inflammation and capacity of muscle regeneration and mitochondrial biogenesis.

The result of the serum ratio of the pro-inflammatory cytokine to anti-inflammatory cytokine showed a tendency to decrease significantly by the GBE + WPH mixture administration, which was found to be due to the increase of serum anti-inflammatory cytokine IL-10 level. IL-10, a cytokine primarily produced by monocytes, suppresses the synthesis of pro-inflammatory cytokines, and it is known that physical exercise increases serum IL-10 level, creating an anti-inflammatory environment [24,25]. Although there were no changes in the serum levels of pro-inflammatory cytokines, a significant increase in anti-inflammatory cytokine created systemic anti-inflammatory circumstances. These changes have a positive effect on skeletal muscle, because inflammation exacerbates muscle atrophy via the STAT and NF-κB pathways [12]. Also, IL-10 is known to prevent age-related inflammation and insulin resistance, which adversely affect muscle [26]. In addition, it could also be the basis for the inference that the GBE + WPH mixture has an exercise-like effect on the body.

A 10-month-old mouse corresponds to an about 40-year-old human, as 10 months is the middle age of a mouse’s lifespan, when the muscle function begins to deteriorate [27]. In terms of the efficacy of the GBE + WPH mixture on skeletal muscle strength and mass, it effectively inhibited the reduction of muscle strength so that grip strength was stably maintained significantly in the M and H groups. The weight of three muscle tissues showed a tendency to increase, and there was a statistically significant difference in the M and H groups. Therefore, it was found that the GBE + WPH mixture was effective in both the IM-induced muscle atrophy mice and aging-induced muscle atrophy mice and the effective dose of the GBE + WPH mixture is over 900 mg/kg. In addition, aging causes a more intensive decrease in fast-twitch fiber than slow-twitch fiber [28], but two fast-twitch fiber muscles (quadriceps and gastrocnemius) significantly increased in all GBE + WPH mixture-administered groups compared to the control group.

As a mechanism study, three aspects of skeletal muscle aging were investigated; (1) balance of muscle protein synthesis and degradation, (2) muscle fiber differentiation, and (3) mitochondrial biogenesis in skeletal muscle. The PI3K/Akt pathway determines muscle mass by regulating muscle protein synthesis and degradation. This pathway is activated by exercise but becomes less active with age, exacerbating muscle atrophy in the elderly [10,29]. The GBE + WPH mixture effectively increased the activity of the PI3K/Akt pathway and inhibited muscle atrophy.

It is known that the expression of MyoD and Myogenin decreases in aged mice’s skeletal muscle as the differentiation capacity of the muscle decreases with aging [30]. Myostatin, which weakens muscle fiber differentiation capacity by suppressing the expression of those factors, also accelerates muscle protein degradation via suppressing the activation of Akt [31]. We practically verified an increase in the expression of MyoD, Myogenin, and activated Akt in contrast to a decrease in myostatin by the GBE + WPH mixture administration. Although we did not directly confirm the change in the number or activity of satellite cells, precursors of skeletal muscle cells, we could speculate that the GBE + WPH mixture positively affected satellite cells through an increase in essential factors for muscular regeneration and an increase in actual muscle mass.

Mitochondria, organelles that produce chemical energy, are related to skeletal muscle strength. As aging progresses, the expression of PGC1a, a representative factor in mitochondrial biogenesis, decreases. This condition increases oxidative stress in skeletal muscle and accelerates sarcopenia [32]. It was experimentally confirmed that the number of mitochondria and grip strength were improved as the expression of PGC1a and the upstream factor of PGC1a, SIRT1, increased. In addition, these results are also expected to be associated with a significant increase in soleus weight. This is because slow-twitch fiber muscle contains a high content of mitochondrial and increased mitochondrial volume or function reduces oxidative stress in muscles that increase with age and suppresses muscle atrophy.

This study attempted to investigate whether the GBE and WPH mixture, which had already confirmed the effect on the IM-induced muscle atrophy model, had the same effect on the aging-induced muscular deterioration model. Consequently, we clearly confirmed the anti-sarcopenic effect of the GBE and WPH mixture on aging mice and identified changes in aging-related mechanisms. Therefore, depending on the clinical trial results along with further studies, the GBE + WPH mixture could be one of the candidates to prevent sarcopenia.

## 5. Conclusions

This study aimed to investigate the effect of the GBE + WPH mixture on age-associated muscular deteriorations in aged mice. Aging causes chronic low-grade inflammation in the body and disrupts the muscle protein synthesis and degradation balance, and reduces the capacity of muscle regeneration and mitochondrial biogenesis. These symptoms cause muscle atrophy and exacerbate sarcopenia, but the GBE + WPH mixture ameliorated these physiological changes. The GBE + WPH mixture suppresses systemic low-grade inflammatory status by increasing serum IL-10 level, and muscle mass and strength were increased via PI3K/Akt pathway activation. In addition, the expression of muscle regeneration and mitochondrial biogenesis-related factors was also significantly increased by the GBE + WPH mixture, contributing to the improvement of muscle function.

## Figures and Tables

**Table 1:** The oligonucleotide primer sequences used in RT-qPCR.

Gene	Sequences	
*16S rRNA*	F: CCGCAAGGGAAAGATGAAAGAC	R: TCGTTTGGTTTCGGGGTTTC
*Hexokinase 2*	F: GCCAGCCTCTCCTGATTTTAGTGT	R: GGGAACACAAAAGACCTCTTCTGG
*Myostatin*	F: ACTGGACCTCTCGATAGAACACT	R: ACTTAGTGCTGTGTGTGTGGAGAT
*Myogenin*	F: TGGTCCCAACCCAGGAGATCATTT	R: ACATATCCTCCACCGTGATGCTGT
*MyoD*	F: CAACTGCTCTGATGGCATGATGG	R: TGTTCTGCATCGCTTGAGGATGTC
*Sirt1*	F: CAAGATGCTGTTGCAAAGGAACC	R: CAAGATGCTGTTGCAAAGGAACC
*PGC1α*	F: AAGTGTGGAACTCTCTGGAACTG	R: GGGTTATCTTGGTTGGCTTTATG
*β-actin*	F: ATATCGCTGCGCTGGTCGTC	R: AGGATGGCGTGAGGGAGAGC
